# Implementing a multi-sectoral response to HIV: a case study of AIDS councils in the Mpumalanga Province, South Africa

**DOI:** 10.1080/16549716.2017.1387411

**Published:** 2017-10-23

**Authors:** Pinky Mahlangu, Jo Vearey, Liz Thomas, Jane Goudge

**Affiliations:** ^a^ Gender and Health Research Unit, South African Medical Research Council, Pretoria, South Africa; ^b^ School of Public Health, Faculty of Health Sciences, University of Witwatersrand, Johannesburg, South Africa; ^c^ African Centre for Migration & Society, School of Social Sciences, University of Witwatersrand, Johannesburg, South Africa

**Keywords:** HIV response, multi-sectoral approach, governance, AIDS councils, Mpumalanga Province, South Africa

## Abstract

**Background**: A multi-sectoral response is advocated by international organisations as a good strategy to address the multiple drivers and impact of human immunodeficiency virus/acquired immune deficiency syndrome (HIV/AIDS), and was historically mandated as a condition of funding. In March 2017, the South African National AIDS Council (SANAC) launched the latest 5 year National Strategic Plan (NSP) to address HIV, sexually transmitted infections and tuberculosis. As with previous iterations, the NSP calls for multi-sectoral action (MSA) and mandates AIDS councils (ACs) at different levels to coordinate its implementation. Efforts have been made to advocate for the adoption of MSA in South Africa, yet evaluation of these efforts is currently limited.

**Objective**: This paper assesses the implementation of a multi-sectoral response to HIV in South Africa, through a case study of the Mpumalanga Province.

**Methods**: We identified and reviewed key policy documents, conducted 12 interviews and held six focus group discussions. We also drew on our involvement, through participant observation, in the development of NSPs and in AC meetings.

**Results**: SANAC is struggling to provide much-needed support to provincial, district and local ACs. Therefore, most ACs are generally weak and failing to implement MSA. Membership is voluntary, there is a lack of sustained commitment and they do not include representatives from all sectors. There is little capacity to undertake the activities necessary for coordinating the implementation of MSA, and unclear roles and responsibilities within ACs result in divisions and tension between sectors. There is inadequate senior political leadership and funding to facilitate effective implementation of MSA.

**Conclusion**: We identified three interventions that we argue are required to support the effective implementation of MSA: strengthening and stabilising the SANAC structure; building capacity of ACs; and creating an enabling environment for effective implementation of MSA through political leadership, support and resourcing of the HIV response.

## Background

Governance is a key and decisive factor in the outcome of efforts to respond to the human immunodeficiency virus/acquired immune deficiency syndrome (HIV/AIDS) epidemic, and critical for the effective implementation of programmes and policies that require coordination across different sectors and levels of government [1,2]. It is increasingly recognised that no single sector can address the multiple drivers and impacts of HIV and AIDS [3,4], and that integrated, multi-level efforts by government, working together with other sectors, including civil society and the private sector, are urgently needed [5,6]. Multi-sectoral approaches to HIV ‘seek to reduce HIV prevalence, provide care and treatment to those living with HIV and AIDS, and mitigate the impact of the epidemic to those affected by employing the appropriate mix of health and non-health based interventions, and *involving a broad array of stakeholders from multiple sectors in their design and implementation*’ [6,p.223]. Drawing together all major stakeholders in society, regardless of their sector, work or organisational affiliation, multi-sectoral action (MSA) aims to create a mechanism for regular information sharing and coordination. Evidence suggests that countries such as Uganda and Senegal have controlled the spread and reduced the prevalence of HIV through MSA [7].

A multi-sectoral approach to HIV was introduced through a number of donor initiatives in sub-Saharan Africa. The World Bank Multi-Country AIDS Programme was launched in 2000, and one of the conditions of the grant was that countries should develop multi-sectoral HIV and AIDS plans [8]. The Global Fund to Fight AIDS, Tuberculosis and Malaria, launched in 2002, also required countries to demonstrate a commitment to MSA as a condition for funding [9]. Both funding organisations required countries to establish multi-sectoral national AIDS councils (NACs), to function as coordinating bodies and oversee the development and implementation of multi-sectoral HIV and AIDS plans [7,10]. At the 2001 United Nations General Assembly Special Session, member countries made a declaration of commitment to develop and implement multi-sectoral national strategies and financing plans to combat HIV [11]. UNAIDS further developed the ‘Three Ones’ principle: one agreed HIV and AIDS action framework that provides the basis for coordinating the work of all partners; one national AIDS coordinating authority, with a broad multi-sector mandate; and one agreed country-level monitoring and evaluation system, with the aim of encouraging concerted action on HIV and AIDS at the national level and harmonising donor interventions [12].

A multi-sectoral approach has since been adopted in the national HIV and AIDS plans of many countries in sub-Saharan Africa. While commendable investments were made in advocating for the adoption of MSA, this has not been matched by efforts to review the successes and challenges of implementing MSA, other than a few notable exceptions in Uganda, Senegal, Tanzania and Rwanda [13–15]. The reviews conducted to date were limited to the framework of the World Bank and the Global Fund, which focused more on compliance and the benefits of MSA than on the experiences of implementation. As a result, these reviews fail to unpack the institutional and process issues that enable – or limit – effective implementation of MSA. Reviews of ACs have also focused at national level with national AIDS commissions, and do not include the sub-national level structures where cross-sectoral work is much more complex [10,16].

In light of this gap, this paper reflects on the process of implementing MSA on the HIV response in South Africa. We document the experiences of ACs at national, provincial, district and local municipality levels in coordinating implementation of a multi-sectoral response to HIV and AIDS during the 2012–2016 period of the South African National Strategic Plan (NSP). The paper explores the factors that enable or hinder effective MSA, and makes recommendations on what can be done to strengthen MSA on the HIV response in South Africa.

In South Africa, multi-sector collaboration – as a central tenant of the national response to HIV – has evolved over time. The National AIDS Plan (NAP), developed under the leadership of the National AIDS Convention of South Africa (NACOSA) in 1994, was the first broad-based and inclusive plan to guide the response to HIV and AIDS in South Africa [17]. NACOSA was a non-governmental organisation (NGO) to which government, the private sector, trade unions and other NGOs, including community- and faith-based organisations, were affiliated [18]. The content of the NAP reflected commitment to MSA, and recommended inclusion of non-state actors in the implementation process, as well as establishment of multi-sectoral coordinating structures to be led by the Presidency at national level, and the Offices of the Premier at provincial level [17]. Responding to HIV and AIDS was, at that time, declared a ‘Presidential-led project’, giving it preferential access to funds under the new Government of National Unity post-1994.

Unfortunately, the recommendations made in the NAP were never implemented. Instead, the response to HIV and AIDS was placed under the ambit of the National Department of Health (NDOH), within a narrow health and biomedical framework, where a National AIDS Programme Director was employed to oversee implementation of the NAP [19]. The nine South African provinces followed the lead from the national government and placed HIV and AIDS within their respective provincial Departments of Health. This process involved reassigning people from elsewhere in the civil service to work as provincial AIDS coordinators, rather than recruiting from the extensive pool of AIDS activists who were believed to have valuable knowledge and understanding about HIV and of the community experiences of HIV [20]. The roles and responsibilities for developing and implementing plans at both national and provincial levels were unclear, with district structures still developing at that time. As a result, implementation was characterised by multiple parallel activities lacking in coherence and continuity within government, and between government and civil society [20]. In 1996, there were allegations of unlawful tendering after the Department of Health awarded a R14.27 million tender for the production of a stage play, Sarafina II, aimed at educating young people about the dangers of HIV and AIDS. In what became known as ‘the Sarafina II debacle’, there were criticisms about the play’s flawed messaging and excessive cost (one-fifth of the national AIDS budget), which led to an investigation by the Public Protector and the Parliamentary Portfolio Committee on Health [21]. Three years later, in 1999, the government’s failure to provide Zidovudine, an early antiretroviral treatment] and announcement of its intention to make HIV a notifiable medical condition increased the tension between government and civil society [22].

**Figure 1.**
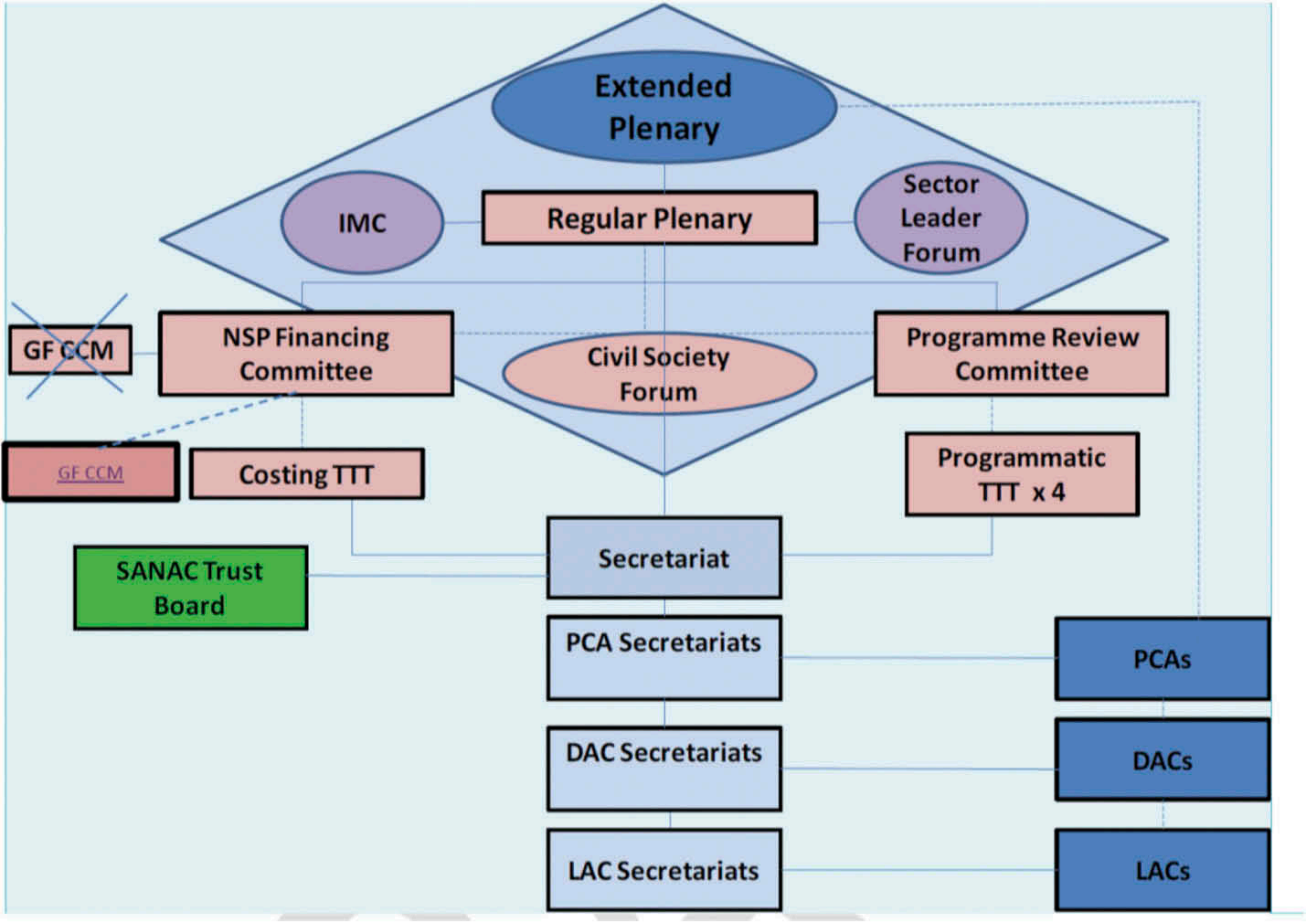
Structure of the South African National AIDS Council (SANAC). IMC, Inter-Ministerial Committee; GF CCM, Global Fund Country Coordinating Committee; NSP, National Strategic Plan; TTT, technical task team; PCA, Provincial Council on AIDS; DAC, district AIDS council; LAC, local AIDS council. (Source: SANAC. Enhanced progress report: national strategic plan draft SANAC Procedural Guidelines. 2016.) [39]

The HIV/AIDS and STI National Strategic Plan for South Africa 2000–2005 reiterated the key principles of cooperation and inclusion that were included in the original NAP (1994–1999), with a focus on establishing a national multi-sectoral coordinating structure, called the South African National AIDS Council (SANAC), to drive programme implementation [23]. SANAC is made up of government, civil society and the private sector (see Figure 1 for the structure of SANAC). It oversees the development of the national multi-sectoral response to HIV and AIDS, and monitors its implementation. The mandate of SANAC includes providing policy advice to government, advocating for multi-sectoral engagement, monitoring implementation of the NSP, creating and strengthening strategic partnerships, mobilising resources for implementation of AIDS programmes, and recommending appropriate HIV and AIDS research [24]. Although this first NSP (2000–2005) expressed a commitment to MSA, the President’s denialism of the aetiology of AIDS, and the Minister of Health’s questioning of the safety and affordability of antiretroviral drugs undermined the government’s ability to mobilise and lead a united response to the epidemic [25]. In response, civil society engaged in mass action and litigation that forced the government to provide antiretroviral therapy and a national Prevention of Mother-to-Child Transmission programme. It was a continuous struggle for the two actors to work together. A review of implementation of the plan highlighted that overall coordination of activities at SANAC and within civil society was a major weakness [26].

**Figure 2.**
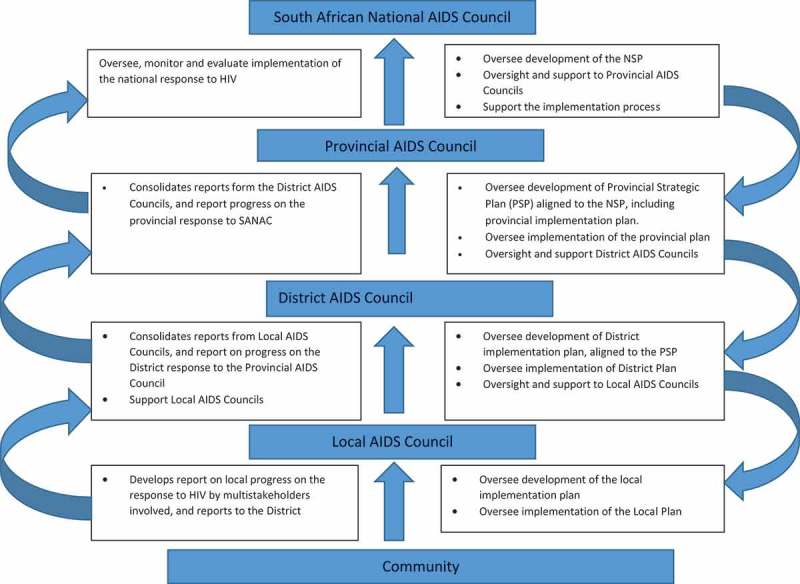
Roles and responsibilities, and relationship between AIDS councils at different levels. SANAC, South African National AIDS Council; NSP, National Strategic Plan; PSP, Provincial Strategic Plan. Source: Developed by the authors.

The second NSP (2007–2011) was widely praised for its innovative content, the result of a comprehensive consultative process which involved a wide range of stakeholders: government, civil society, academic and research institutions, labour, business and regional representatives from UNAIDS [27]. This consultative processes provided some opportunities for reconciliation between government and civil society. Wouter (2010) defined the period between 2007 and 2011 as a new era of boldness and leadership in relation to HIV. The temporary absence of the Minister of Health due to ill health, and the key role played by the Deputy Minister of Health at the time, an advocate of universal access to antiretrovirals, created an opportunity for dialogue between government and civil society. There was an underlying principle in the plan that government alone will not be able to develop and implement a comprehensive and successful response to HIV and AIDS, hence the focus on engaging other sectors outside government. The concept ‘MSA’ was formally referred to and incorporated in the plan, which called for ‘a comprehensive national multi-sectoral response to HIV and AIDS’ [28,p.7,9,19). All government departments and sectors of civil society were required to use the NSP as the basis to develop their own HIV and AIDS strategic and operational plans in order to achieve a focused, coherent, country-wide response to HIV and AIDS [23]. For the first time, provinces, districts and local government were given the critical role of acting as implementing agencies of the NSP. The envisaged model was that the national level multi-sectoral structure (SANAC) would be replicated at provincial, district and local levels to achieve effective and coordinated programme implementation [28]. An AC at any level should provide a platform and facilitate collaboration between sectors, with an intention to improve synergies, reduce fragmentation of action, and decrease duplication of HIV programmes and interventions aimed towards meeting the goals of the NSP [28]. As with SANAC, provincial, district and local AIDS councils (PACs, DACs and LACs, respectively) were meant to be comprised of members representing civil society, government and the private sector [29,30]. Figure 2 explains the roles and responsibilities, and describes the relationship between SANAC, PACs, DACs and LACs.

While this paper analyses the third NSP (2012–2016), South Africa now has a new NSP in place (2017–2022). This new (fourth) NSP is not different from the NSP reviewed in this paper: both the third and fourth NSPs are based on the principle of MSA, and in both plans ACs are central in realising the ideal of MSA; they are mandated to coordinate a multi-sectoral response to HIV and AIDS at provincial, district and local levels. The new NSP (2017–2022) talks about the need to ‘formally establish and restructure AIDS Councils, to ensure that they are empowered and strengthened to provide effective coordination’ [31]. While the new NSP has pointed out this strategic goal, there is no strategy or plan of what will be done to realise the goal. Recommendations made in this paper also address this gap.

## Methods

### Study setting

The study was conducted with ACs at national, provincial, district and local municipality levels, using the Mpumalanga Province (MP) as a case study. The MP is located in the eastern part of South Africa, bordering both Swaziland and Mozambique, and is divided into three district municipalities: Gert Sibande, Ehlanzeni and Nkangala. All three districts were included in the study to gain an in-depth overview of the response to HIV and AIDS in the province. The district municipalities had between five and seven local municipalities, but only two local municipalities from each of the three districts were included in this study (Umjindi and Nkomazi from Ehlanzeni District, Emalahleni and Victor Khanye from Nkangala District, and Chief Albert Luthuli and Msukaligwa from Gert Sibande District). The selection was based on their availability and willingness to participate.

### Study design

Using an exploratory case study design, the study involved both primary data collection conducted between March and September 2013, and secondary data analysis. We reviewed HIV and AIDS policy documents and key reports, and conducted primary data collection using participant observations, focus group discussions (FGDs) and key informant interviews (KIIs). While this paper draws from the data collected in 2013, its findings are still relevant and consistent with some of the challenges highlighted in the recent review conducted in the province.

### Data collection

A retrospective review of nine policy documents was undertaken, including the review of the NSP for HIV, tuberculosis (TB) and sexually transmitted infections (STIs) 2012–2016, the Provincial Strategic Plan for HIV AIDS, STIs and TB (2012–2016), and the Strategic Implementation Plans of two DACs and three LACs. One DAC had a draft implementation plan which was excluded from the review, and the three remaining LACs did not have implementation plans available at the time of data collection. We also reviewed progress and financial reports reflecting on the implementation of the NSP 2012–2016 at provincial, district and local levels. The document review guide was developed based on the objectives of the study, and explored how MSA is defined and conceptualised; the structural arrangements at different levels; implementation plans including costing, reporting, monitoring and evaluation; and successes and challenges reported in implementation of a national multi-sectoral response to HIV.

Semi-structured KIIs were conducted with 12 people: a representative from SANAC, the HIV manager from the PAC, HIV coordinators from two DACs, HIV coordinators from six LACs, and two technical advisors who were supporting two districts at the time [funded by Deutsche Gesellschaftfür Internationale Zusammenarbeit (GIZ) and the USAID Sexual HIV Prevention Programme (SHIPP)]. A purposive sampling strategy was used to select people with knowledge about the development, implementation and coordination of a multi-sectoral response, and how ACs operate at different levels.

We conducted six FGDs with members of the PAC, one DAC and four LACs, and the number of participants ranged between four and 12 people. All members of the ACs were invited to the FGDs, but only those who were available self-selected to participate. The FGDs were facilitated by the first author and conducted in isiZulu and Isiswati. We used an open-ended FGD guide to enable reflection on the structure of ACs, to explore the participants’ understanding of a multi-sectoral approach to HIV, to understand how the ACs operate and the environments within which they operate, and to reflect on challenges and opportunities enabling or restricting effective coordination of a multi-sectoral response. Participant observation was undertaken in public consultation meetings during the development of the NSP (2012–2016), and in AC meetings at provincial, district and local levels to understand the content of the members’ discussions. We were also involved in the consultative process leading to the development of the current NSP (2017–2022). The first author participated in public consultation meetings hosted by SANAC, and highlighted the challenges experienced by ACs and the need to strengthen them going forward into the new NSP (2017–2022). Digitally recorded FGDs and KIIs were transcribed verbatim and translated into English by the first author.

### Data analysis

The analysis of data adopted an inductive approach to undertake a thematic content analysis, guided by the research question and objectives of the study [32,33]. To analyse data from the document review, KIIs and FGDs, we developed a table following Walt and Gilson’s framework [34] to understand the actors, content, process and context within which coordination and implementation of a multi-sectoral approach to HIV may be undertaken. The process of deriving themes from interviews and FGDs involved detailed reading and rereading of the transcripts to identify recurring themes and patterns emerging from the data [35]. All transcripts were read by PM, and a sub-sample of transcripts was read by LT. After discussion, a coding frame was developed and the transcripts were coded by PM. If new codes emerged, they were discussed with LT, and a decision was made about how they fitted within the coding frame. Data management was done manually, and all transcripts were printed and colour-coded according to the emerging categories.

## Results

### Composition of AIDS councils

Most DACs and LACs did not consist of representatives from all three sectors. Civil society was the most represented sector across all ACs included in the study. Examples of civil society organisations participating in ACs include community-based organisations (e.g. Mothers to Mothers and sex workers), faith-based organisations (e.g. pastors and traditional healers forums) and NGOs (e.g. Anova Health and All Seasons Home Based Care). There was limited participation from government departments and a notable absence of the private sector:

You find that sometimes the private sector is working far away from other sectors [in silos], it is still difficult to bring in the private sector even after several attempts to invite them, and some departments. Mainstreaming of HIV is still a problem in some departments. (FGD member of the DAC)

Membership in the ACs is voluntary, with no financial benefit for participation, resulting in a lack of commitment and a membership that is continuously changing. There are no systems to support involvement and continued participation, a frustration shared by many interviewed:

We get tired of working in a non-paying job [volunteering]. There is no [financial] reward for participating in the AIDS council. You do this out of the goodness of your heart. Remember, most of us are not employed, we are volunteers even in the organisations we represent in the LAC. It can be tiresome. (FGD, member of LAC representing civil society)

### Operating procedures

We found no evidence of guidelines to inform the establishment of ACs, other than a mention in the NSP (2012–2016) [39,p.62] that ‘there will be a clear guiding framework to support implementation and set out expected roles and responsibilities’. While some ACs have sub-committees that are aligned to the objectives of the NSP (prevention, treatment care and support, orphans and vulnerable children, and human rights), others have no sub-committees. Only two of the six LACs and one of the three DACs included in the study had sub-committees tasked with achieving specific objectives. While some AC meetings were planned in advance, some happened in an *ad hoc* fashion, meeting only if the need arose, and mainly driven by the municipal HIV and AIDS programme. The review of documents showed that the highest number of meetings that ACs had had was three times a year. Some ACs, however, had only met once or twice in the previous year, and some meetings took place in an *ad hoc* manner.

Some of the activities documented in the minutes of the various AC meetings included arranging HIV counselling and testing campaigns, compiling reports from members regarding implementation of a varied scope of HIV and AIDS programmes (people living with HIV/AIDS, orphans and vulnerable children, and key populations) in their organisations, preparing for events including World AIDS Day, making inputs on treatment guidelines, reporting on challenges in communities, reflecting on functionality of the structures, and discussing funding issues and planned activities and how members can collaborate.

The effectiveness of the meetings depends on the people participating, and their ability to make decisions, implement resolutions, and report back to the AC. (KII with MPAC HIV coordinator)

Without decision-making power in their own organisation, AC members were unable to suggest different ways of working to improve the synergies with other organisations in the AC. Moreover, some organisations were sending different people to each meeting:

Organisations keep sending new faces in meetings. That delays progress and can be a waste of time, spent updating the new people on the resolution of the previous meeting before going through the agenda of that day. Often the person who has attended that time cannot even report on some of the action items that were allocated to their organisation in the previous meeting. (KII with district HIV coordinator)

### Capacity to coordinate MSA

All ACs had appointed HIV coordinators who were employed and paid by the respective municipality. HIV coordinators played a critical role as the secretariat of the ACs, with a responsibility to drive the process, bring members together, facilitate and oversee the development of the implementation plan with activities and time frames aligned to the NSP, monitor and evaluate progress on implementation of the plan, and report to the higher level structure. Yet they experienced many challenges. Except for two of the HIV coordinators interviewed in this study, most had limited capacity to perform in their role. Most of them were junior, and did not have the necessary skills and confidence needed to effectively design and deliver robust HIV activities.

Some of them don’t even have grade 12, and know nothing about HIV in local government. They couldn’t even make an input during the provincial meetings that we had with them. (KII with provincial HIV coordinator)

Some HIV coordinators have no background and knowledge on HIV and AIDS. They are political appointees with contracts linked to a five-year term of the mayor in office. (KII with district HIV coordinator)

With few notable exceptions, other members of the ACs at the local level did not have the capacity to undertake critical tasks required in coordinating a multi-sectoral response.

… the problem is the level of capacity in most AIDS councils. Some of them [members], especially from civil society, do not have much education. Things like writing strategic plans and reports are technical activities, which are difficult [for them] to do. The HIV coordinator has to do all the work. (KII with district technical advisor)

Despite these challenges, good practice examples were noted by two ACs which had HIV coordinators who were reported to be making a positive impact:

She makes things happen. She is very passionate about her work. If something needed to be approved and the manager seem not to act, she would take a chair and sit down by the door of the manager, when the manager enters or exit the office she will be there. She would tell the manager ‘people are dying out there’, the manager will be left with no choice but to sign for whatever that she needs him to sign or approve. (KII with technical advisor)

In some instances, the AC ‘ceases to function after the HIV coordinator’s contract ends, and he or she leaves the municipality with all information about the AIDS council’ (KII with district HIV coordinator), owing to a heavy reliance on the one person.

### Relations between sectors

The following account describes the state of the relationship between sectors:

Civil society is not taken seriously because it operates in a different way from government. Their [civil society’s] contribution and ideas most often not taken on board in meetings. (KII with district technical advisor)

Another respondent spoke about power issues:

Most of the financial resources for implementation of HIV and AIDS activities comes from government. Civil society mainly contributes people who implement the programmes funded by government, USAID, and others. I think that paints a master–servant relationship which leads government to act like they are better than others because they have financial power. (KII with district technical advisor)

There was also a concern raised about failure to fulfil expected roles, which created mistrust. One participant explained:

Working with government has been frustrating. You go and mobilise people, and make efforts to convince them to go and test and seek health care. They go to the clinics and find no services, and come back to you complaining. There is nothing you can do if government does not play its role. (KII with member of DAC)

This suggests that the relationship between government and civil society was characterised by divisions and scepticism.

### Institutional challenges within the NAC

Our findings show that the national multi-sectoral structure (SANAC) is undergoing a number of institutional challenges, including organisational restructuring and revitalisation, which are characterised by contestation and changes in leadership positions [36,37]. ‘Since its establishment in 2002–2011, the SANAC Secretariat has been relying on financial and administrative support from the Department of Health, and just recently establishing itself as an independent institution’ [38,p.100]. There is no clarity regarding roles and responsibilities between the Office of the Deputy President, the SANAC Secretariat, and the Department of Public Service and Administration (key HIV and AIDS coordinating divisions).

As a result, there is a lack of guidance and support from the national level to support and develop ACs to operate optimally:

The SANAC Secretariat is in process of revitalisation, and currently has one person providing support to provincial AIDS councils. There [has] been a lot of things [discussions] around building the structures [sub-national-level structures], which are currently not doing so well, even the team here at national level is meant to focus on that. (KII with member of the SANAC Secretariat)

SANAC has been caught up in strengthening itself as an organisation and has neglected its oversight role as a national structure. As a result, ACs at sub-national levels have struggled to function effectively.

### Political leadership and support

The level of political leadership and support on the HIV response is different across ACs. The Deputy President of the country during the third NSP (2012–2016), Cyril Ramaphosa, has been actively involved as the Chairperson of SANAC. His leadership role was also evident in public platforms, where he spoke openly about HIV and AIDS, and advocated for MSA [38]. In contrast, there was a lack of political leadership in some districts and some LACs. While some HIV coordinators were political appointees, strategically located in the office of the mayor, others were located in the Community Service department, and struggled to get much-needed political support from the mayor. They described the mayor who ‘is never there, and always has other issues taking precedence, other than HIV and AIDS issues’ (KII with LAC HIV coordinator).

The lack of involvement of political leaders has left a vacuum, which has compromised ACs. Participants spoke about the effects of a lack of political support, highlighting that:

No one listens to you if the invite or request does not come with endorsement from the mayor. (KII with district HIV coordinator)

However, two ACs were found to have the necessary support from the mayor:

Through the mayor’s involvement, we mobilised stakeholders to participate in the AIDS council, and also brought investors who funded some activities of the AIDS council. (KII with LAC HIV coordinator)

One of the HIV coordinators narrated a positive story of a supportive political leader, highlighting some of what the AC was able to achieve in such an environment:

Our municipality has for three consecutive years been awarded as the best performing AIDS council in the province, and we would not have done it without his leadership and support. (KII with LAC HIV coordinator)

Another participant observed:

I once worked at [district X]. When there is something needing action, the mayor ensures that it become a success. She is very hands-on, very much involved. She supports the coordinator, very much so, that’s what makes them to succeed. (KII with technical advisor)

### Financial resources

The central issue highlighted at the provincial level was that the activities listed in the implementation plan were unfunded and, as a result, likely to remain a wish list. Members of LACs spoke about the variation in the budgets allocated to ACs by district and local municipalities, emphasising that some municipalities do not prioritise HIV and AIDS in their budgets.

HIV and AIDS [activities] then becomes an unfunded mandate. (FGD with member of LAC)

Budget constraints at local level had led some members of the AC to use money from their pocket for the activities of the AC:

We sometimes have to use our own money for transport to travel to some of the wards which are some distance apart from each other because the AC does not have a budget. (FGD with member of LAC)

Other issues identified at a national level included inaccurate cost estimation and budgeting, and poor tracking of the contribution of the private sector [39].

## Discussion

Contrary to Putzel’s argument that the multi-sectoral coordinating structures were a ‘faulty, imposed organisational template’ [40], South Africa has willingly adopted MSA as one of the key principles of the national response to HIV [14]. However, its implementation has been difficult. Similarly to other studies, we have found that ACs experience a number of challenges limiting their capacity to effectively coordinate a multi-sectoral response to HIV in South Africa [30,41]. Our data show that the lack of stability and structure at SANAC does not allow for its effective support of sub-national structures: the PACs, DACs and LACs. As a result, ACs at all levels, with a few exceptions, are weak and struggle to perform their mandate. The lack of representation of some sectors in DACs and LACs contradicts the purpose for which ACs were established, resulting in poor coordination and omission of the work of stakeholders from multiple sectors. The informal structure of ACs, based on volunteers with continuously changing membership, does not allow for undertaking a complex and continuous task of coordination. Volunteerism can be associated with a lack of accountability, lack of ownership, low morale and lack of commitment [42].

In the absence of a framework to guide their development and responsibilities, the roles of AC members are unclear, and there are no rules and regulations governing the way in which they operate. This is a critical element that needs to be addressed to ensure that ACs operate with strategic direction [42,43]. AC meetings are supposed to be used as a platform for coordination, with clear plans on how to achieve the goals of the NSP, strategies to implement the plans, and a framework to monitor and evaluate progress. However, most of the AC meetings are ineffective, with agenda items that are not geared towards coordination. This partly explains the lack of participation of senior people, who could perceive the meetings to be a waste of time. We found limited evidence of a few HIV coordinators who undertake the critical components involved in coordination such as bringing sectors together and reporting on the progress and work of the AC. We argue that, in the main, the limited capacity of most HIV coordinators in DACs and LACs limits effective coordination of a multi-sectoral response to HIV. Based on the evidence that MSA depends on relationships of mutual trust, shared understanding and interests, and joint accountability [44], the existing poor relations and divisions between sectors found in our study present a major obstacle to developing an effective multi-sectoral response to HIV in South Africa.

Our study has highlighted the lack of an enabling environment for ACs to effectively coordinate implementation of MSA at district and local levels. Without political support, it has been difficult for ACs to mobilise stakeholders and the resources needed to facilitate the process of coordination. While political leaders at the local level in South Africa are expected to chair ACs, there are no punitive measures to enforce compliance. Some political leaders have wrongfully used ACs as public platforms for publicity and to campaign for political support, and coordinating structures for once-off events (e.g. World AIDS Day), rather than permanent structures to be supported throughout the year. Moreover, political leaders still struggle to integrate HIV in the municipal planning process, resulting in limited budgets allocated for HIV programmes.

The successful experience of Uganda and Senegal has clearly highlighted the importance of political leadership and support in a multi-sectoral response to HIV. In both Uganda and Senegal, the government had little to lose and everything to gain in taking early action on AIDS. The donor community was willing to provide assistance if government demonstrated commitment to the campaign against HIV and AIDS. The political leaders shunned myths associated with HIV and AIDS, and they listened and acted on scientific medical advice. Political action at the highest level of government was critical in ensuring that all government departments are involved in the response to HIV and AIDS. The epidemic was put beyond the partisan politics in Uganda. No one could occupy a public office without demonstrating a commitment in the response to HIV. Government not only opened space for civil society to participate, but also played a crucial role in convincing the sector to get involved, and ensured that they understood their mandate and contribution in the response to HIV [14].

We believe that MSA on the response to HIV is both necessary and possible in the South African context. Given the institutional challenges highlighted among the ACs included in our study, we make the following recommendations to help improve coordination of a multi-sectoral HIV response. First, ACs at local level would benefit from national guidance and oversight from SANAC, creating an enabling environment for effective coordination. SANAC’s support to ACs should include facilitating a process to build capacity of PACs, DACs and LACs, and development of a framework with guidelines to inform the establishment and operation of ACs. The guidelines should make it a requisite that ACs have multi-sectoral representation, and that membership consists of people with decision-making powers, who will be formally appointed as members of ACs, and their commitment enforced through the signing of membership contracts, which are critical for continuity. Secondly, ACs need training on the critical elements required for effective coordination, including strategic planning, monitoring and evaluation, and reporting on progress at the local level. While the need for strengthening SANAC has been pointed out in previous reviews, we suggest, thirdly, the strengthening of governance and accountability through development of a framework that will stipulate which division within SANAC will be responsible to build capacity of ACs at local level, and what indicators will be used to assess the quality of support provided. The existing poor relations between government and the civil society sector should be managed, encouraging tolerance and respect, and helping sectors to recognise the interconnections and interdependency among them [45]. ACs could participate in a team-building process facilitated by SANAC. The process could be undertaken for a short-term period of 4–6 weeks, with regular monitoring to assess and help ACs to address issues as they arise. The need to expand efforts to improve political commitment and support on the HIV response is reiterated in this study. Such efforts should focus on finding ways to incentivise and recognise commitment, and to develop measures to enforce commitment where it does not exist.

The findings of this study should be viewed in light of several limitations. The case study approach used in this study is not a representation of all nine provinces, and therefore findings cannot be generalised across all ACs in other provinces. Generalisation of case study findings is limited to the case itself [46]. With all qualitative enquiry, the analysis is influenced by subjective interpretation, but all efforts were made in this study to remain true to the data [47].

In conclusion, this paper describes the factors limiting the effective implementation of a multi-sectoral approach to the HIV and AIDS response of six DACs and three LACs in the Mpumalanga Province of South Africa. It fills a gap in the literature, pointing broadly to what is needed for an effective multi-sectoral response to HIV and AIDS. While MSA promises to be a good strategy for collaborative action to address the multiple drivers and impact of HIV and AIDS, its coordination through the currently weak ACs is problematic. Three levels of intervention are proposed to address the challenges experienced by ACs. First, there is an urgent need to strengthen and stabilise the SANAC structure to be able to assume its guiding and oversight role. Secondly, SANAC should facilitate the process of building the capacity of PACs, DACs and LACs to ensure effective coordination of the multi-sectoral response to HIV and AIDS at local level, and help to address the poor relations between sectors. Thirdly, an enabling environment for the implementation of MSA should be created through political commitment and support for MSA, providing ACs with the resources needed for effective coordination of a multi-sectoral response to HIV and AIDS.

## Paper context

Multi-sectoral action (MSA) is advocated as a good strategy to address the multiple drivers and impact of HIV and AIDS. Reflection on the process of implementing MSA has been scanty, conducted in some African countries but not in South Africa, and with limited focus at local level. This study shows that MSA is not a panacea and that its implementation is challenging. Organisational templates imposed by external agencies are not always practical and need to be adopted with caution, considering the context and what is possible at local level.
